# When Large-Scale Assessments Meet Data Science: The Big-Fish-Little-Pond Effect in Fourth- and Eighth-Grade Mathematics Across Nations

**DOI:** 10.3389/fpsyg.2020.579545

**Published:** 2020-09-30

**Authors:** Ze Wang

**Affiliations:** Department of Educational, School & Counseling Psychology, University of Missouri, Columbia, MO, United States

**Keywords:** big-fish-little-pond effect, data science, latent variable modeling, large-scale assessment, R, TIMSS

## Abstract

The programming language of R has useful data science tools that can automate analysis of large-scale educational assessment data such as those available from the United States Department of Education’s National Center for Education Statistics (NCES). This study used three R packages: EdSurvey, MplusAutomation, and tidyverse to examine the big-fish-little-pond effect (BFLPE) in 56 countries in fourth grade and 46 countries in eighth grade for the subject of mathematics with data from the Trends in International Mathematics and Science Study (TIMSS) 2015. The BFLPE refers to the phenomenon that students in higher-achieving contexts tend to have lower self-concept than similarly able students in lower-achieving contexts due to social comparison. In this study, it is used as a substantive theory to illustrate the implementation of data science tools to carry out large-scale cross-national analysis. For each country and grade, two statistical models were applied for cross-level measurement invariance testing, and for testing the BFLPE, respectively. The first model was a multilevel confirmatory factor analysis for the measurement of mathematics self-concept using three items. The second model was multilevel latent variable modeling that decomposed the effect of achievement on self-concept into between and within components; the difference between them was the contextual effect of the BFLPE. The BFLPE was found in 51 of the 56 countries in fourth grade and 44 of the 46 countries in eighth grade. The study provides syntax and discusses problems encountered while using the tools for modeling and processing of modeling results.

## Introduction

Data science tools, particularly those developed with the statistical language of R (R Core Team, 2020), have been increasingly used in educational and social sciences. For scholarly articles, R is the second most frequently used data science software following SPSS (Muenchen, n.d.). Given its integrated system of data wrangling, statistical modeling, visualization, and communication (Grolemund and Wickham, 2018), R is appealing to those conducting empirical analysis (i.e., using real data) as well as those interested in simulation studies. Currently, there are over 16,000 R packages available on the Comprehensive R Archive Network (CRAN) – R’s main repository of packages – and more packages in other repositories (such as GitHub). Packages are developed for various topics (for example, see “Task Views” at the CRAN). They, together with R’s core packages, provide tools for researchers to work with different aspects of using data. There are also search engines (e.g., RSeek, Nabble), online communities (e.g., Stack Overflow, Cross Validated, RStudio Community), and mailing lists (e.g., R-help, R-devel) that are available for additional help for using R. At the same time, the sheer amount of R resources seems daunting to beginner users, let alone its sometimes unfamiliar or non-user-friendly ways of “doing” things.

Large-scale assessments (LSAs) are great data sources (Rutkowski et al., 2014). An LSA typically involves complex design frameworks for the development of items, sampling participants, data collection, and variable creation. The United States Department of Education’s National Center for Education Statistics (NCES) houses multiple international LSA studies across the lifespan from early childhood to adults (National Center for Education Statistics, n.d.). These studies are sponsored by two organizations: The International Association for the Evaluation of Educational Achievement (IEA) and the Organization for Economic Cooperation and Development (OECD), although the work is typically directed by testing firms and research institutes in cooperation with national research institutions and governmental agencies. In the United States, the National Assessment of Educational Progress (NAEP) is an LSA that was first conducted in 1969 (National Center for Education Statistics, 2020). LSAs allow researchers to use nationally and internationally representative data to answer research questions and even for policymaking (Wagemaker, 2014).

Despite its rich data, LSAs have been used only to the extent that is far from its potential (Wang, 2017). It takes quite some time for one to get familiar with an LSA. Substantive researchers may be unaware of the relevant content in LSAs that can be used for their research. Or, they may lack the expertise to go through the database which may contain hundreds of datasets, or to run large-scale analysis. At the same time, when a researcher does use an LSA, many times only data from a single or a few countries/regions are used for analysis (e.g., Wang et al., 2012; Smith et al., 2020).

In this article, I illustrate how to use a few R packages that I have found particularly useful for conducting large-scale cross-national analysis using NCES data. Those packages are EdSurvey (Bailey et al., 2020), MplusAutomation (Hallquist and Wiley, 2018), and tidyverse (Wickham et al., 2019). Several other packages were used for this study but the main functions are from these three packages.

The goal of this study is twofold. First, it examines and continues to document the big-fish-little-pond effect (BFLPE) using the Trends in International Mathematics and Science Study (TIMSS), an international LSA by IEA. Second, it demonstrates the implementation of data science tools to carry out large-scale cross-national analysis. I provide syntax so that interested readers can replicate the analysis. The syntax can also be modified for similar analyses.

### A Few Data Science Tools

Traditional tools tend to treat different aspects of the whole data manipulation and statistical analysis in compartments. Each tool is for a special purpose and the user has to piece all the different elements together to use multiple tools for a more complex problem. To illustrate my point, think about the different statistics courses a doctoral student in educational psychology typically takes. The student may take courses that cover regression, analysis of variance, factor analysis, structural equation modeling, etc. For those courses, the professor may provide data for homework problems and/or projects, or the student may be encouraged to work on projects with their “own” data. In the latter case, the data may be from the student’s advisor or another fellow graduate student. Most likely, the data are already cleaned/managed in the sense that the variables are ready to be used to apply the learned statistical techniques. The student may be unappreciative of the data management steps that lead to the cleaned data until they are involved in a bigger grant project or at the dissertation/thesis stage. However, data wrangling is time-consuming. Data scientists spend from 50 to 80% of their time collecting and preparing unruly digital data (Lohr, 2014). With the amount of data available in every field, the toolbox of quantitative researchers, especially those working with empirical data, needs to include tools that allow them to handle various types and quantities of data.

#### R and RStudio

The programming language R has increasingly become a popular statistical analysis software. It is open source meaning that everyone can access and contribute to its development. Despite its relatively long history (The first publicly available version of R was released in 2000), R has only gained more acceptance among social science researchers in the past decade or so. R was born out of S, which was intended to be a programming language focused on data analysis, and has evolved into a system used not only by computer programmers and data analysts but also by physical scientists, psychologists, journalists, etc. Researchers use R because (a) it is free and open-source; (b) it has many packages built to meet various needs of statistical analysis; (c) there are freely provided useful resources among the R community; (d) collaboration using R is easy; (e) analysis with R can be highly reproducible; and (f) data wrangling using R can be fast, dependable, and highly replicable (Barrett, 2019).

RStudio is an integrated development environment (IDE) for R. It uses R to develop codes and analysis that can be executed and has greater usability than R. Essentially RStudio can be thought of as the interface between the user and R. It depends on and adds onto R, which means that the R program has to be installed before RStudio for RStudio to implement R. Any R package or function can be used in RStudio. RStudio has many features for good usability. One basic feature I particularly like is auto-completion. When the user types the first few characters of an R command, function, or the name of a data object that has been created, RStudio will show a list of complete names from which the user can choose to insert. This saves a lot of time typing and finding typos. For more experienced users who would like to develop their own packages, RStudio provides tools that automatically organize the structure necessary for package development. Interested readers can check out the “Advanced R” book (Wickham, 2019).

#### EdSurvey

One particular challenge of using LSAs is to access and browse the data. A researcher may have some idea about the LSA after reading its description online or the user’s guide, but getting hands-on with the data usually means downloading big zipped files, unzipping them, and making them viewable using statistical software such as SPSS or SAS. Sometimes, there are hundreds of datasets that can be explored. The R EdSurvey package, recently developed by Bailey et al. (2020), makes accessing and transforming LSAs data to be R-ready a breeze.

EdSurvey was developed for data downloading, processing, manipulation, and analysis of LSAs by NCES and incorporates special survey methodologies (complex sampling, sampling weights, replicate weights, etc.) in a single package. In addition to data procuration, EdSurvey has methods developed for statistical analysis. However, these methods are for analysis of observed variables. Researchers interested in using latent variable modeling techniques such as factor analysis has to rely on other packages. The R package lavaan (Rosseel, 2012) is widely used for latent variable modeling but its capabilities are still limited for analysis using LSAs. For example, sampling weights in lavaan can only be used for non-clustered data. Although it is possible to use lavaan for multilevel structural equation modeling, only listwise deletion can be used for handling missing data.

#### MplusAutomation

Mplus (Muthén and Muthén, 1998-2017) is a comprehensive program for structural equation modeling (SEM) including latent variable modeling. Mplus is especially popular among applied researchers. It is syntax-based but relatively easy to use. It has many capabilities for advanced analysis (e.g., multilevel latent variable modeling, intensive longitudinal data analysis, Bayesian analysis) and can handle many data issues (e.g., missing data, non-normality, clustered data, complex survey designs); new methodologies are routinely added for its development. The most recent version is Version 8. Unlike R, it is not open source, and the user purchases licenses for the software and technical support services.

One drawback of Mplus is that the input or output of every model is stored as a separate file (.inp for input files and .out for output files). If one is to run many models, extracting information from the many output files can be a problem. The process can be tedious and error-prone. In addition, while Mplus is great for modeling, it has very limited capability for data management either to prepare data for the model or to further process data contained in the output files. To address these problems, Hallquist developed the R MplusAutomation package that can create, batch run, and extract results from many models (Hallquist and Wiley, 2018). Data to be analyzed can be managed in R like other R objects; sections of the Mplus input syntax are embedded in the object created by calling the mplusObject function; the mplusModeler function creates Mplus input files as well as dataset files if requested; the runModels function runs a group of Mplus models; and the Mplus output files (i.e., those with.out extension) can be extracted using the readModels. In addition, the MplusAutomation package provides functions to tabulate summary statistics, compare models, and extract parameters.

#### Tidyverse

Another useful R package for programming large-scale analysis with LSAs is tidyverse (Wickham et al., 2019). Technically, tidyverse is not a single R package; rather, it is a collection of R packages that share an underlying design philosophy, grammar, and data structures, which makes data wrangling, analysis, and visualization relatively easy.

For cross-national analysis using data from LSAs, it is necessary to process data before and after modeling them. While it is possible to use other packages (e.g., the “data.table” package; Dowle and Srinivasan, 2019) or the R base package to get the same results, I chose tidyverse because of its comprehensiveness and because it is relatively easy to use. The functions used in the present study are a tiny part of all the capacity of tidyverse. Here I would like to particularly point out the pipe operator (%>%) and the map function. The pipe operator comes from the magrittr (Bache and Wickham, 2014) package but is loaded automatically with tidyverse. It chains sequential operations to avoid creating intermediate objects and nested function calls and to make the syntax more readable. The map function is from the purr package (Henry and Wickham, 2020a) which is also loaded automatically with tidyverse. It takes a vector and a function as function inputs (i.e., arguments), applies the function to each element of the vector, and returns the results in a list of the same length. It is an efficient way of eliminating “for” loops so that the code is easier to write and read. If the output is more desired in a vector format, there are four variants which return a specific type of results: map_lgl (for a logical vector), map_int (for an integer vector), map_dbl (for a double vector), and map_chr (for a character vector).

### The Big-Fish-Little-Pond Effect

When students compare their ability in an academic subject, they tend to compare themselves in their immediate context. As a result, students in higher-achieving contexts have lower self-concept than similarly able students in lower-achieving contexts. This phenomenon is called the big-fish-little-pond effect (BFLPE; Marsh, 1990). The BFLPE can be explained by the social comparison theory. According to this theory, individuals evaluate themselves by comparing themselves to others (Festinger, 1954; Suls et al., 2002). For such comparisons, those in an individual’s immediate social group often serve as the comparison target (Rogers et al., 1978). To evaluate one’s academic ability, the student may compare his/her academic position with their classmates when they form their academic self-concept. As a result, students from different classes may have different self-evaluations even when their academic abilities are the same.

Due to its social comparison nature, the BFLPE is a contextual effect, which occurs when the aggregate of a person-level characteristic (e.g., mathematics ability) is related to the outcome (e.g., mathematics self-concept) even after controlling for the effect of the individual characteristic; in other words, the “context” has an additional effect on the individual. Contextual effects can be examined using multilevel modeling statistical techniques (Raudenbush and Bryk, 2002). In a two-level modeling framework (e.g., students nested within classes), if the predictor variable is grand-mean centered, the between-level effect is the contextual effect; if the predictor is group-mean centered, the difference between the between-level effect and the within-level effect is the contextual effect.

### TIMSS 2015

TIMSS is an international assessment of student achievement in mathematics and science in fourth and eighth grades. It is sponsored by IEA and directed by the TIMSS & PIRLS International Study Center at Boston College. The first TIMSS was administered in 1995 and has been administered every 4 years since then. TIMSS 2015 was the sixth cycle and is the most recent administration with data released to the public (TIMSS 2019 results are expected to be released in December 2020). In addition to tests measuring achievement, background and non-cognitive information is collected from students, teachers, and school principals, allowing researchers to examine relationships between achievement and personal and contextual factors across countries/regions.

Large-scale assessments have been used to study the BFLPE across countries. Marsh and Hau (2003) used the Program of Student Assessment (PISA) 2000 data collected in 26 countries; Seaton et al. (2009) used PISA 2003 data collected in 41 countries; Nagengast and Marsh (2011) used PISA 2006 data and examined the BFLPE with a total international sample from 57 countries, a total United Kingdom sample, and four samples from United Kingdom counties. Using TIMSS 2007, Wang (2015) examined the BFLPE in 49 countries in eighth-grade mathematics. Wang and Bergin (2017) further examined the BFLPE in 59 countries using TIMSS 2011 in eighth-grade mathematics. However, no study has investigated the BFLPE across many countries using TIMSS 2015.

## Materials and Methods

### Samples, Variables, and Data

The present study used TIMSS 2015 data from 56 countries at the fourth-grade level and 46 countries at the eighth-grade level (Foy, 2017). The total sample consisted of 330,204 students from 15,740 classes in 10,964 schools in fourth grade and 285,190 students from 11,856 classes in 8,500 schools in eighth grade (see Tables 1, 2).

**TABLE 1:** Results of Model 1 in Fourth Grade.

									Within		Between			
				χ^2^					variance		variance		ICC	
										
Country	^#^Schools	^#^Classes	^#^Students	Est.	p	CFI	TLI	RMSEA	Est.	*p*	Est.	*p*	Est.	*p*
Abu Dhabi, United Arab Emirates	163	219	5001	6.07	0.05	0.99	0.97	0.021	0.332	<0.001	0.023	<0.001	0.065	<0.001
Buenos Aires, Argentina	136	292	6435	2.21	0.33	1.00	1.00	0.004	0.638	<0.001	0.043	<0.001	0.062	<0.001
Dubai, United Arab Emirates	168	316	7453	1.30	0.52	1.00	1.00	0.000	0.454	<0.001	0.048	<0.001	0.095	<0.001
United Arab Emirates	558	891	21177	5.70	0.06	1.00	0.99	0.009	0.348	<0.001	0.034	<0.001	0.090	<0.001
Armenia	148	234	5384	6.81	0.03	1.00	0.99	0.022	0.724	<0.001	0.047	0.004	0.061	0.003
Australia	287	498	6057	2.63	0.27	1.00	1.00	0.007	0.628	<0.001	0.009	0.071	0.015	0.070
Belgium (Flemish)	153	295	5404	36.99	0.00	0.99	0.97	0.057	0.758	<0.001	0.023	0.001	0.030	<0.001
Bulgaria	149	233	4228	0.19	0.91	1.00	1.00	0.000	0.629	<0.001	0.097	<0.001	0.134	<0.001
Bahrain	182	345	8575	0.97	0.62	1.00	1.00	0.000	0.084	0.010	0.005	0.040	0.057	0.003
Canada	441	696	12283	2.34	0.31	1.00	1.00	0.004	0.666	<0.001	0.030	0.001	0.042	<0.001
Chile	179	179	4756	0.10	0.95	1.00	1.00	0.000	0.612	<0.001	0.043	<0.001	0.066	<0.001
ON, Canada	151	271	4574	1.58	0.45	1.00	1.00	0.000	0.669	<0.001	0.034	0.007	0.049	0.002
QC, Canada	121	152	2798	7.71	0.02	0.99	0.98	0.032	0.638	<0.001	0.024	0.034	0.036	0.032
Cyprus	148	243	4125	7.01	0.03	1.00	0.99	0.025	0.660	<0.001	0.036	<0.001	0.052	<0.001
Czechia	159	265	5202	50.40	0.00	0.98	0.93	0.068	0.626	<0.001	0.009	0.090	0.014	0.081
Germany	204	213	3948	1.91	0.38	1.00	1.00	0.000	0.676	<0.001	0.017	0.012	0.025	0.012
Denmark	193	194	3710	0.60	0.74	1.00	1.00	0.000	0.697	<0.001	0.025	0.001	0.034	0.001
England	147	176	4006	2.69	0.26	1.00	1.00	0.009	0.615	<0.001	0.040	<0.001	0.061	<0.001
Spain	358	379	7764	6.38	.04	1.00	0.99	0.017	0.632	<0.001	0.039	<0.001	0.058	<0.001
Finland	158	300	5015	2.41	0.30	1.00	1.00	0.006	0.670	<0.001	0.015	0.025	0.022	0.025
France	164	273	4873	53.12	0.00	0.97	0.91	0.073	0.615	<0.001	0.022	0.004	0.034	0.002
Georgia	153	188	3919	8.56	0.01	0.99	0.96	0.029	0.342	<0.001	0.029	0.013	0.078	0.003
Hong Kong SAR	132	145	3600	0.55	0.76	1.00	1.00	0.000	0.666	<0.001	0.021	0.005	0.030	0.005
Croatia	163	223	3985	2.60	0.27	1.00	1.00	0.009	0.597	<0.001	0.017	0.008	0.028	0.007
Hungary	144	241	5036	4.09	0.13	1.00	1.00	0.014	0.704	<0.001	0.021	0.012	0.030	0.012
Indonesia	230	312	8319	2.60	0.27	1.00	1.00	0.006	0.255	<0.001	0.097	0.001	0.277	<0.001
Ireland	149	214	4344	1.81	0.40	1.00	1.00	0.000	0.678	<0.001	0.011	0.088	0.017	0.082
Iran, Islamic Rep. of	248	291	7928	0.46	0.80	1.00	1.00	0.000	0.381	<0.001	0.038	0.005	0.090	<0.001
Italy	164	257	4373	102.62	0.00	0.93	0.79	0.108	0.626	<0.001	0.019	0.009	0.030	0.010
Jordan	254	272	7861	3.05	0.22	0.99	0.98	0.008	0.026	0.326	0.004	0.379	0.138	0.003
Japan	148	148	4383	0.62	0.73	1.00	1.00	0.000	0.587	<0.001	0.020	0.003	0.033	0.003
Kazakhstan	171	239	4702	0.44	0.80	1.00	1.00	0.000	0.489	<0.001	0.078	<0.001	0.138	<0.001
Korea, Rep. of	149	188	4669	1.42	0.49	1.00	1.00	0.000	0.733	<0.001	0.042	<0.001	0.054	<0.001
Kuwait	166	294	7296	0.08	0.96	1.00	1.00	0.000	0.076	0.003	0.015	0.016	0.161	<0.001
Lithuania	225	290	4529	4.86	0.09	1.00	0.99	0.018	0.692	<0.001	0.013	0.040	0.018	0.044
Morocco	358	374	10428	2.05	0.36	1.00	1.00	0.002	0.325	<0.001	0.118	<0.001	0.267	<0.001
Northern Ireland	118	153	3116	0.35	0.84	1.00	1.00	0.000	0.655	<0.001	0.019	0.050	0.028	0.044
Netherlands	129	223	4515	8.91	0.01	1.00	0.99	0.028	0.765	<0.001	0.003	0.430	0.004	0.430
Norway (4th grade)	139	219	4164	1.44	0.49	1.00	1.00	0.000	0.550	<0.001	0.032	0.002	0.055	0.001
Norway	140	222	4329	1.64	0.44	1.00	1.00	0.000	0.656	<0.001	0.017	0.030	0.025	0.028
New Zealand	174	459	6322	8.67	0.01	1.00	0.99	0.023	0.645	<0.001	0.028	<0.001	0.041	<0.001
Oman	300	353	9105	1.58	0.45	1.00	1.00	0.000	0.025	0.777	0.002	0.781	0.060	0.145
Poland	150	254	4747	29.55	0.00	0.99	0.97	0.054	0.643	<0.001	0.030	0.013	0.045	0.008
Portugal	217	321	4693	0.19	0.91	1.00	1.00	0.000	0.599	<0.001	0.042	<0.001	0.066	<0.001
Qatar	211	224	5194	8.23	0.02	0.99	0.97	0.025	0.235	<0.001	0.016	<0.001	0.065	<0.001
Russian Federation	208	217	4921	5.33	0.07	1.00	1.00	0.018	0.539	<0.001	0.022	0.001	0.039	<0.001
Saudi Arabia*	189	189	4337	6.77	0.03	0.97	0.90	0.024	–0.135	0.256	–0.018	0.275	0.120	0.003
Singapore	179	358	6517	39.19	0.00	0.98	0.95	0.053	0.602	<0.001	0.137	<0.001	0.185	<0.001
Serbia	160	192	4036	0.85	0.65	1.00	1.00	0.000	0.595	<0.001	0.036	0.001	0.056	<0.001
Slovak Republic	198	327	5773	18.39	0.00	0.99	0.96	0.038	0.620	<0.001	0.033	0.001	0.051	<0.001
Slovenia	148	255	4445	1.61	0.45	1.00	1.00	0.000	0.621	<0.001	0.016	0.014	0.025	0.013
Sweden	144	211	4142	2.73	0.25	1.00	1.00	0.009	0.633	<0.001	0.019	0.008	0.029	0.008
Turkey	242	251	6456	2.56	0.28	1.00	1.00	0.007	0.497	<0.001	0.040	<0.001	0.074	<0.001
Chinese Taipei	150	177	4291	2.45	0.29	1.00	1.00	0.007	0.658	<0.001	0.019	<0.001	0.028	<0.001
United States	250	497	10029	21.54	.00	0.99	0.98	0.032	0.630	<0.001	0.032	<0.001	0.049	<0.001
South Africa	297	298	10932	3.54	0.17	1.00	0.99	0.009	0.285	<0.001	0.027	0.004	0.087	<0.001

CFI, comparative fit index; TLI, Tucker–Lewis index; RMSEA, root mean square error of approximation; ICC, intraclass correlation coefficient. *model run with 10 random sets of starting values.

**TABLE 2:** Results of Model 1 in Eighth Grade.

									Within		Between			
				χ^2^					variance		variance		ICC	
										
Country	^#^Schools	^#^Classes	^#^Students	Est.	p	CFI	TLI	RMSEA	Est.	*p*	Est.	*p*	Est.	p
Abu Dhabi, United Arab Emirates	156	208	4838	3.02	0.22	1.00	1.00	0.010	0.503	<0.001	0.049	<0.001	0.089	<0.001
Buenos Aires, Argentina	128	138	3253	1.60	0.45	1.00	1.00	0.000	0.748	<0.001	0.054	<0.001	0.067	<0.001
Dubai, United Arab Emirates	135	264	6149	1.62	0.44	1.00	1.00	0.000	0.573	<0.001	0.060	<0.001	0.095	<0.001
United Arab Emirates	477	763	18012	1.84	0.40	1.00	1.00	0.000	0.529	<0.001	0.055	<0.001	0.094	<0.001
Armenia	145	228	5060	0.86	0.65	1.00	1.00	0.000	0.906	<0.001	0.058	<0.001	0.060	<0.001
Australia	285	645	10338	31.77	0.00	0.99	0.97	0.039	0.600	<0.001	0.099	<0.001	0.142	<0.001
Bahrain	105	197	4918	3.23	.20	1.00	0.99	0.011	0.112	0.166	0.009	0.194	0.072	<0.001
Botswana	159	169	5964	2.47	.29	1.00	1.00	0.006	0.453	<0.001	0.046	<0.001	0.092	<0.001
Canada	276	409	8757	13.80	0.00	1.00	0.99	0.026	0.648	<0.001	0.077	<0.001	0.107	<0.001
Chile	171	173	4849	5.30	0.07	1.00	1.00	0.019	0.706	<0.001	0.033	<0.001	0.044	<0.001
ON, Canada	138	217	4520	3.41	0.18	1.00	1.00	0.013	0.666	<0.001	0.063	<0.001	0.086	<0.001
QC, Canada	122	175	3950	8.92	.01	1.00	0.99	0.030	0.649	<0.001	0.094	<0.001	0.126	<0.001
Egypt	211	215	7822	2.23	0.33	1.00	1.00	0.004	1.586	0.279	0.152	0.330	0.087	<0.001
England	143	213	4814	55.00	0.00	0.98	0.93	0.075	0.508	<0.001	0.160	<0.001	0.239	<0.001
Georgia	153	187	4035	4.82	0.09	1.00	0.99	0.019	0.544	<0.001	0.041	<0.001	0.070	<0.001
Hong Kong SAR	133	145	4155	2.19	0.33	1.00	1.00	0.005	0.684	<0.001	0.038	<0.001	0.053	<0.001
Hungary	144	241	4893	4.42	0.11	1.00	1.00	0.016	0.711	<0.001	0.054	<0.001	0.070	<0.001
Ireland	149	204	4704	6.53	0.04	1.00	1.00	0.022	0.654	<0.001	0.031	<0.001	0.045	<0.001
Iran, Islamic Rep. of	250	251	6130	2.71	0.26	1.00	1.00	0.008	0.669	<0.001	0.066	<0.001	0.089	<0.001
Israel	200	200	5512	0.25	0.88	1.00	1.00	0.000	0.632	<0.001	0.061	<0.001	0.088	<0.001
Italy	161	230	4481	0.53	0.77	1.00	1.00	0.000	0.692	<0.001	0.038	<0.001	0.052	<0.001
Jordan*	252	260	7865	5.31	0.07	0.99	0.98	0.015	–0.067	0.470	–0.005	0.470	0.071	<0.001
Japan	147	147	4745	1.43	0.49	1.00	1.00	0.000	0.641	<0.001	0.012	0.006	0.018	0.006
Kazakhstan	172	239	4887	1.17	.56	1.00	1.00	0.000	0.540	<0.001	0.071	<0.001	0.116	<0.001
Korea, Rep. of	150	170	5309	34.13	0.00	0.99	0.97	0.055	0.850	<0.001	0.017	0.011	0.020	0.011
Kuwait	168	191	4503	1.48	0.48	1.00	1.00	0.000	0.227	<0.001	0.027	<0.001	0.105	<0.001
Lebanon	138	185	3873	9.06	0.01	0.99	0.96	0.032	0.608	<0.001	0.031	0.025	0.048	0.019
Lithuania	208	252	4347	3.78	0.15	1.00	1.00	0.014	0.719	<0.001	0.044	0.001	0.058	<0.001
Morocco	345	375	13035	2.10	0.35	1.00	1.00	0.002	0.151	0.472	0.010	0.479	0.061	<0.001
Malta	48	223	3817	3.43	0.18	1.00	1.00	0.014	0.632	<0.001	0.108	<0.001	0.146	<0.001
Malaysia	207	326	9726	8.87	0.01	0.99	0.98	0.019	0.514	<0.001	0.060	<0.001	0.104	<0.001
Norway (8th grade)	142	216	4795	0.45	0.80	1.00	1.00	0.000	0.686	<0.001	0.023	0.002	0.033	0.002
Norway	143	216	4697	9.45	0.01	1.00	0.99	0.028	0.731	<0.001	0.021	0.003	0.028	0.002
New Zealand	145	377	8142	6.23	0.04	1.00	0.99	0.016	0.613	<0.001	0.048	<0.001	0.072	<0.001
Oman	301	356	8883	1.42	0.49	1.00	1.00	0.000	0.167	<0.001	0.016	0.001	0.087	<0.001
Qatar	131	238	5403	1.58	0.45	1.00	1.00	0.000	0.418	<0.001	0.037	<0.001	0.081	<0.001
Russian Federation	204	221	4780	7.35	0.03	1.00	0.99	0.024	0.546	<0.001	0.032	<0.001	0.055	<0.001
Saudi Arabia	143	149	3759	43.17	0.00	0.92	0.77	0.075	0.000	1.000	0.000	1.000	0.093	1.000
Saudi Arabia*				12.18	0.00	0.98	0.94	0.037	–0.746	<0.001	–0.062	<0.001	0.077	0.006
Singapore	167	334	6116	0.51	0.77	1.00	1.00	0.000	0.705	<0.001	0.072	<0.001	0.093	<0.001
Slovenia	148	217	4257	4.78	0.09	1.00	1.00	0.018	0.665	<0.001	0.016	0.006	0.024	0.005
Sweden	150	206	4090	15.73	0.00	1.00	0.99	0.041	0.666	<0.001	0.039	<0.001	0.056	<0.001
Thailand	204	213	6482	1.05	0.59	1.00	1.00	0.000	0.544	<0.001	0.077	<0.001	0.124	<0.001
Turkey	218	220	6079	16.90	0.00	1.00	0.99	0.035	0.692	<0.001	0.056	<0.001	0.075	<0.001
Chinese Taipei	190	191	5711	4.93	0.09	1.00	1.00	0.016	0.744	<0.001	0.025	<0.001	0.032	<0.001
United States	246	534	10221	30.98	0.00	1.00	0.99	0.038	0.558	<0.001	0.093	<0.001	0.143	<0.001
South Africa	292	328	12514	18.26	0.00	0.99	0.97	0.026	0.475	<0.001	0.080	<0.001	0.143	<0.001

CFI, comparative fit index; TLI, Tucker–Lewis index; RMSEA, root mean square error of approximation; ICC, intraclass correlation coefficient. *model run with 10 random sets of starting values.

Mathematics self-concept was measured by three items in each grade: (a) I usually do well in mathematics; (b) I am just not good at mathematics (for fourth-graders)/Mathematics is not one of my strengths (for eighth-graders); and (c) I learn things quickly in mathematics. This conceptualization of mathematics self-concept is consistent with Wang (2015); Wang and Bergin (2017) but differs from other articles using TIMSS data such as Marsh et al. (2014, 2015), which included a perceived relative standing item, *Mathematics is more difficult for me than for many of my classmates* for eighth-grade. A similar item in fourth grade is *Mathematics is harder for me than for many of my classmates.* Wang and Bergin (2017) argued that the perceived relative standing item should be separated from the self-concept items.

The three mathematics self-concept items were rated on a 1 to 4 Likert-scale (1 = Agree a lot, 2 = Agree a little, 3 = Disagree a little, 4 = Disagree a lot) and positively worded items were reverse coded so that a higher value corresponded to a higher level of self-concept. Mathematics self-concept was modeled as a latent variable with the three items as indicators and decomposed as having a within and between components during statistical modeling.

TIMSS databases use matrix sampling for the design of test administration where each student answered some but not all items on the test. Student achievement was estimated using item response theory together with a multiple imputation technique. Each student’s mathematics achievement was measured by five plausible values. Those plausible values are not appropriate for reporting individual achievement and are suitable for estimating group characteristics (Wu, 2005). When used for statistical analysis, the five plausible values are treated as multiply imputed values: the analysis is run five times, each time using a single plausible value, and the five sets of results are then combined for point estimates and statistical inference (Enders, 2010).

Data collected in each country are hierarchical because schools were selected first and then classes were selected within schools and either all or sampled students responded to the student survey and the achievement test. For the three-sampling-stage process, TIMSS used probability proportional to size (PPS) sampling to select schools, classes, and students so that schools with more students had a higher probability of being selected and each individual student in the population had roughly the same probability of being selected. Probability weights and adjustment variables for non-responses were calculated for each sampling stage. For analysis using data from each country, a two-level modeling technique was adopted: the within-level was the student level and the between-level was the class level, further clustering at the school-level was accommodated at the between-level by incorporating the probability weights and adjustment factors of selecting schools.

### Statistical Modeling

**FIGURE 1:**
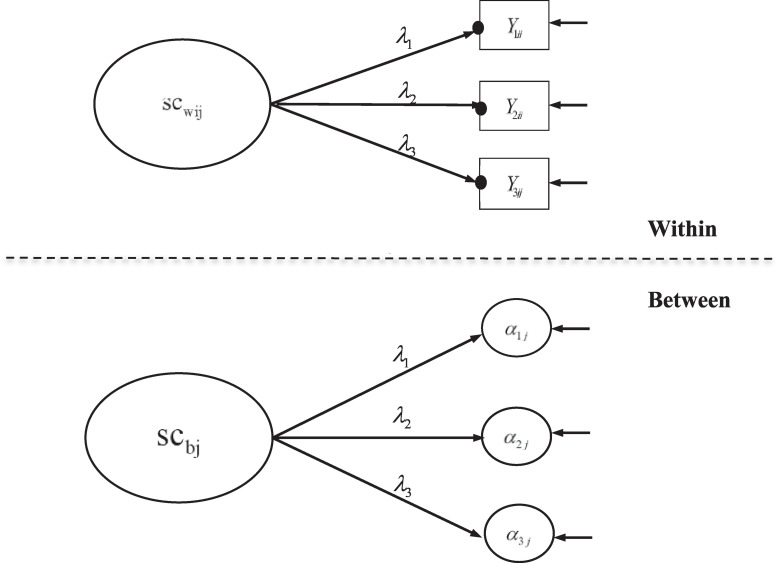
Statistical Model 1 – two-level confirmatory factor analysis model with multilevel measurement invariance of mathematics self-concept. The solid dots indicate random intercepts for different classes. Reprinted with permission from Wang and Bergin (2017); Copyright 2017 by Elsevier.

**FIGURE 2:**
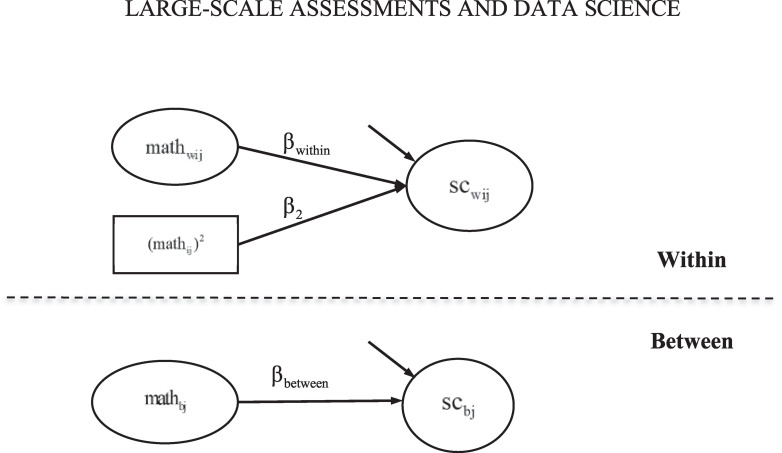
Statistical Model 2 – Model to test the big-fish-little-pond effect. Indicators of the within- and between-level mathematics self-concept are not shown in figure. Reprinted with permission from Wang and Bergin (2017); Copyright 2017 by Elsevier.

Two statistical models are used corresponding to the first two models in Wang and Bergin (2017). The first statistical model is the multilevel confirmatory factor analysis (CFA) model, which was applied for cross-level measurement invariance testing and separately for each country and grade (see Figure 1). The second statistical model is the multilevel SEM model where there are within and between effects of mathematics achievement on mathematics self-concept (see Figure 2). The rescaled difference between the between and within effects is the BFLPE.

To illustrate the models, let **Y_ij_** be a vector with three elements, representing values of the three mathematics self-concept items and let sc_ij_ be the latent mathematics self-concept for student *i* in class *j*. In a single-level CFA model, **Y_ij_** is the vector of indicators of sc_ij_. In the two-level model, sc_ij_ is decomposed into a within and a between component.

(1) $${\text{sc}}_{\text{ij}}={\text{sc}}_{\text{wij}}+{\text{sc}}_{\text{bj}}$$

where sc_wij_ is the within component and sc_bj_is the between component. sc_wij_ and sc_bj_ each is measured by three indicators as shown in equations (2) and (3), respectively.

(2) $${\text{Y}}_{\mathrm{ij}}=\alpha_{j}+\lambda \,{\text{sc}}_{\text{wij}}+{\text{e}}_{\mathrm{ij}}$$

(3) $$\alpha_{j}=\gamma +\lambda \,{\text{sc}}_{\text{bj}}+{\text{r}}_{j}$$

α_**j**_ represents class-specific indicator intercepts at the within level that function as indicators of the latent factor sc_bj_ at the between level; γ is a vector of constants representing the grand mean indicator intercepts at the between level. λ is a vector of factor loadings that are invariant across levels. The invariance of cross-level factor loadings ensures that the interpretation of mathematics self-concept at the within and between levels is the same.

The predictor, mathematics achievement, is also decomposed into a within and a between components.

(4) $${\text{math}}_{\text{ij}}=\mu_{m\,a\,t\,h}+{\text{math}}_{\text{wij}}+{\text{math}}_{\text{bj}}$$

math_ij_ is the mathematics achievement of student *i* in class *j*; μ_*math*_ is a constant representing the grand mean of mathematics achievement for all students in all classes; math_wij_ is student *i*’s mathematics achievement around the class-average mathematics achievement; and math_bj_ is the average mathematics achievement for class *j*, around the grand mean.

Further, the relationship between mathematics self-concept and mathematics achievement is modeled at the two levels in equations (5) and (6), respectively.

(5) $${\text{sc}}_{\text{wij}}=\beta_{w\,i\,t\,h\,i\,n}\,{\text{math}}_{\text{wij}}+\beta_{2}\,{\left({\text{math}}_{\text{ij}}\right)}^{2}+\varepsilon_{i\,j}$$

(6) $${\text{sc}}_{\text{bj}}=\beta_{b\,e\,t\,w\,e\,e\,n}\,{\text{math}}_{\text{bj}}+\delta_{j}$$

In equation (5), the quadratic component of student mathematics achievement is included following previous BFLPE research (e.g., Marsh and Hau, 2003). The standardized effect size of the BFLPE can be calculated as:

(7) $${\text{ES}}_{\text{BFLPE}}=2\times \left(\beta_{b\,e\,t\,w\,e\,e\,n}-\beta_{w\,i\,t\,h\,i\,n}\right)\times \sqrt{\mathrm{Var}\,\left({\mathrm{math}}_{\text{bj}}\right)}/\sqrt{\mathrm{Var}\,\left({\mathrm{sc}}_{\text{wij}}\right)+\mathrm{Var}\,\left({\mathrm{sc}}_{\text{bj}}\right)}$$

Var(math_bj_), *Var*(*sc*_wij_), and *Var*(*sc*_bj_) are the variances of the between-level mathematics achievement, the within-level self-concept, and the between-level self-concept, respectively. The detailed calculations of those variances are illustrated in Wang and Bergin (2017).

### The Syntax

Here I explain the R syntax to examine the BFLPE using TIMSS 2015 data. First, Mplus and R are installed. I also recommend RStudio be installed. All syntax is written using RStudio as an R script (R scripts are like text files). Next, start R or RStudio and install the packages EdSurvey, MplusAutomation, and tidyverse. I also install the rlang package (Henry and Wickham, 2020b) for two functions related to expressions that are used in extracting model fit indices. After the packages are installed, load them using the library function. Packages only need to be installed once on the computer. However, they have to be loaded every time R or RStudio is started.


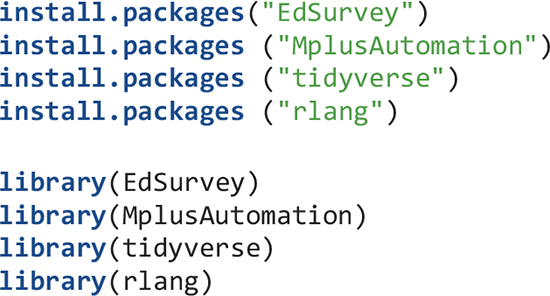


For large-scale analysis with many files, it is important to have a good file system. All related files for the present study are stored in the folder called “BFLPE study.” This folder is created manually in the C: drive and set as the working directory. Alternatively, one can create an R project using RStudio and associate the R project with this working directory. Except for this main folder, all other folders and their contents are created by running syntax in RStudio.

Under this “BFLPE study” folder, there are three subfolders called, “TIMSS,” “Mplus_g4,” and “Mplus_g8,” respectively. The “TIMSS” folder has a subfolder named “2015” inside which are TIMSS 2015 datasets downloaded via an internet connection, as well as META files and text files to be created to facilitate fast reading of data using the EdSurvey package. The “Mplus_g4” folder has two subfolders: “Model1” and “Model2,” corresponding to the two statistical models. “Model 1” includes all Mplus input, output, and data files used for the first statistical model (i.e., multilevel CFA) for all countries at the fourth-grade level and “Model 2” has all Mplus input, output, and data files for the second statistical model (i.e., the BFLPE model) for all countries at the fourth-grade level. The “Mplus_g8” folder also has two subfolders “Model1” and “Model2” with similar information but for eighth grade.


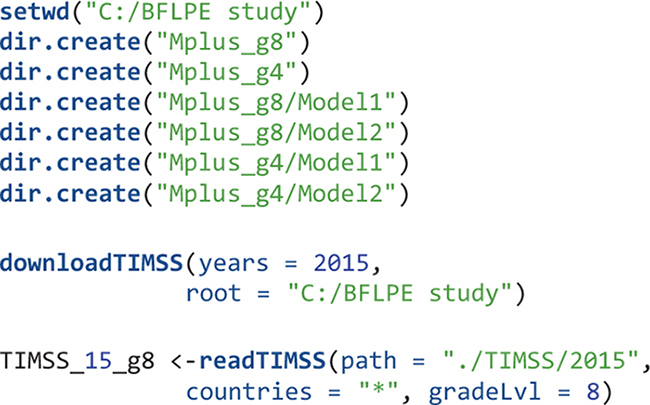


The object TIMSS_15_g8 is a survey data frame (SDF) which stores all TIMSS 2015 information from the student survey, teacher survey, school survey, as well as achievement information in eighth grade for all participating countries. For the remainder of this section, only syntax relevant to eighth-grade analysis is presented. Interested readers can easily modify the syntax to suit for fourth grade.

For this study, students’ mathematics achievement, the three items measuring their mathematics self-concept, and weight variables and adjustment factors accounting for the PPS sampling are used for analysis. Clustering within classes and schools are considered; country id and student id variables are specified as auxiliary variables just for quality control purposes (i.e., to make sure the data are created and used correctly).

The mathematics self-concept items in the SDF are stored as factors and need to be converted to numeric variables. Missing values are specified. All observed variables are standardized within each country. Weight variables and the square term of mathematics achievement used in the Mplus input files are created. For statistical model 1 (i.e., the multilevel CFA model), mathematics achievement data are not used so there is a single dataset for each country. For statistical model 2 (the BFLPE model), each plausible value of mathematics achievement is stored in a different dataset for a total of five datasets for each country. These five datasets are used as imputed datasets in the Mplus syntax.

For analysis, two mathematics self-concept items have to be reverse coded.


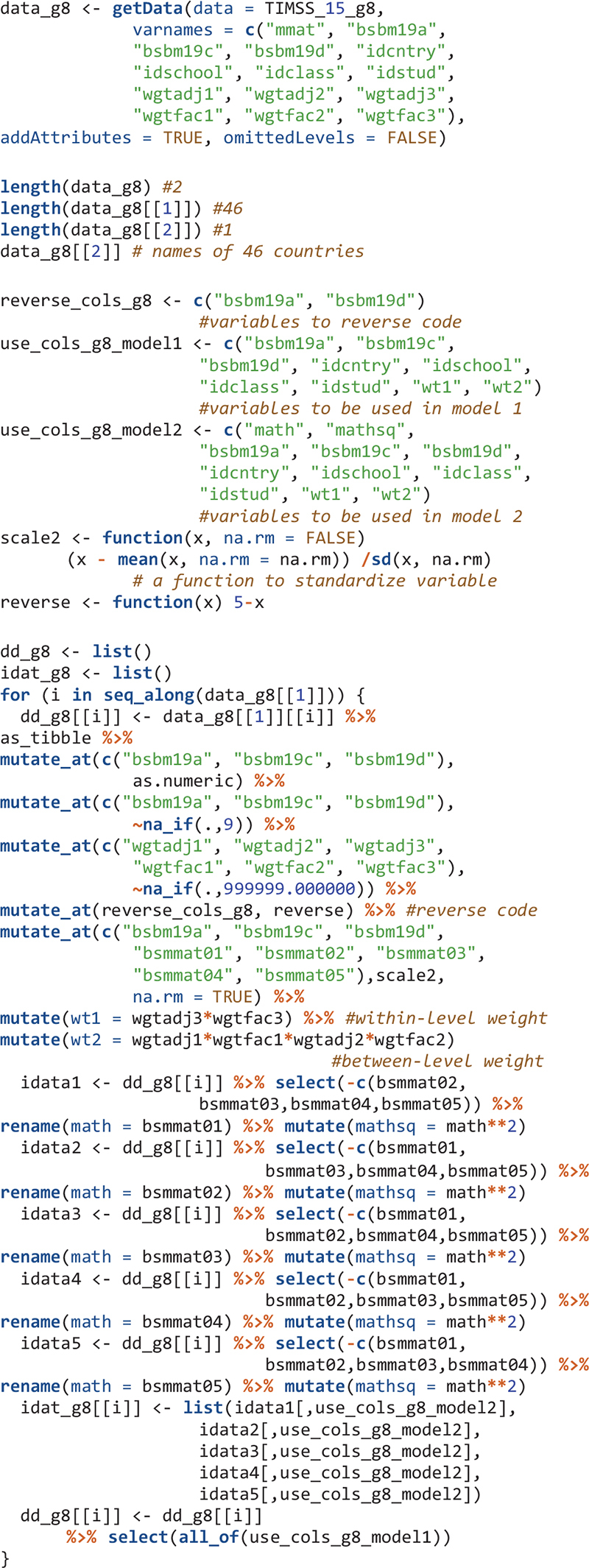


For each of the 46 countries, an object is created – using the mplusObject function – that contains all syntax sections needed to create a Mplus input file for Model 1 for that country. Next, the Mplus input file is created and run using the mplusModeler function. Iterations on countries are done using the map function. Datasets are exported and Mplus output files are created when the model is run. The readModels function extracts information in all Mplus output files in the folder.

The next step after reading Mplus output files is usually to get some type of summary tables. However, for large-scale analysis using LSAs, oftentimes, the model for a few countries may not run properly. In that case, functions such as Summary Table and paramExtract of the MplusAutomation package will not work well and will give errors. Country 22 was the problematic one. Here I simply skip the Mplus output for this country and will manually revise the Mplus input file for this country later. To skip country 22’s Mplus output, I change its Mplus output file extension to.didnotrun so that this file would not be read using the readModels function. I also calculate the number of schools, classes, and students in each country.

The readModels function imports results into R as mplus.model objects with a predictable structure. This structure, shown in Table 3 of Hallquist and Wiley (2018), serves as a guide to what can be extracted from Mplus outputs.


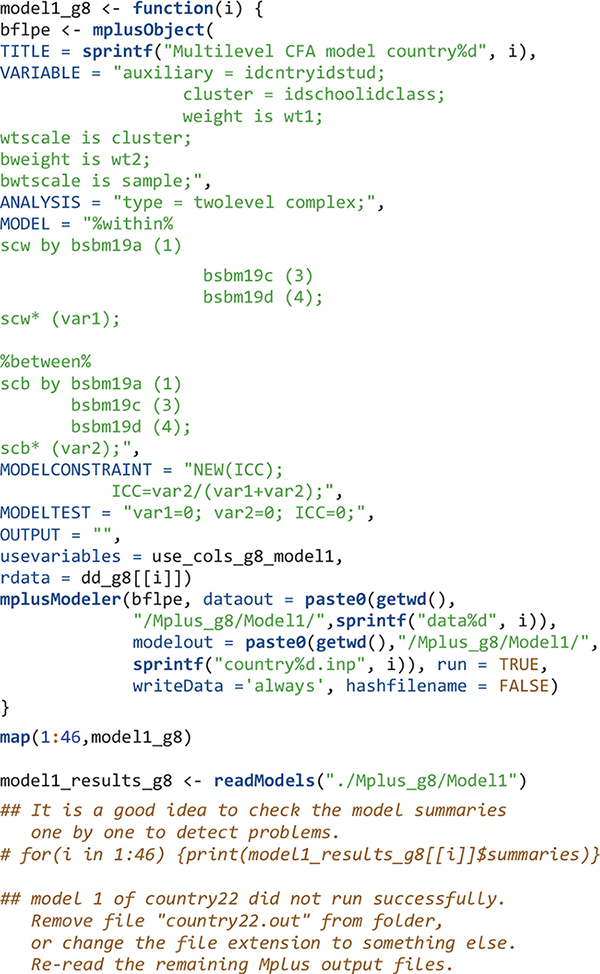



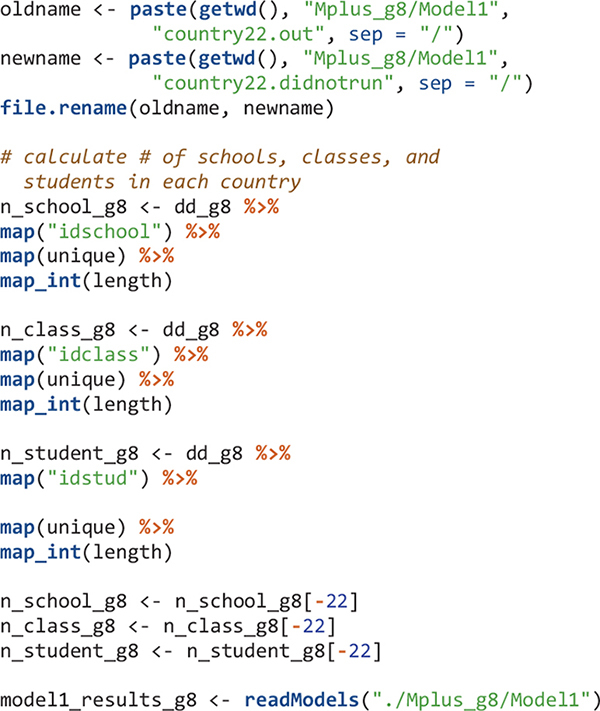


There are multiple summary and fit indices for the modeling results in each country. Extract such information can be easily done by applying the map function and its variants. To extract parameters, we need to know the position of the parameters in the results. For example, after viewing the model1_results_g8[[1]]$parameters$unstandardized object, the within-level variance of mathematics self-concept is in the fourth row. Its estimate and the p value of the estimate are extracted. All results for Model 1 are in the model1_table_g8 object.

The order of elements in R objects is important to match results for countries. The elements in edsurvey.data.frame.list objects in this study (e.g., TIMSS_15_g8 and data_g8) are in ascending order using three-letter country codes. Therefore, the first element is for Abu Dhabi, United Arab Emirates with country code “aad” and the second element is for Buenos Aires, Argentina using country code “aba.” When the Mplus input and output files are created, I simply name them by their country number; therefore the Mplus input and output for Abu Dhabi, United Arab Emirates are country1.inp and country1.out, respectively; and the Mplus input and output for Buenos Aires, Argentina are country2.inp and country2.out, respectively. When reading Mplus outputs using the readModels function, the order in the resulted mplus.model object (the “model1_results_g8”) is ascending alphabetically. Therefore, the first element in “model1_results_g8” has information for country1 and the second element has information for country10 (Chile) instead of country2. We reordered the elements in the mplus.model objects to be ascending according to country numbers (1, 2, 3, etc.).


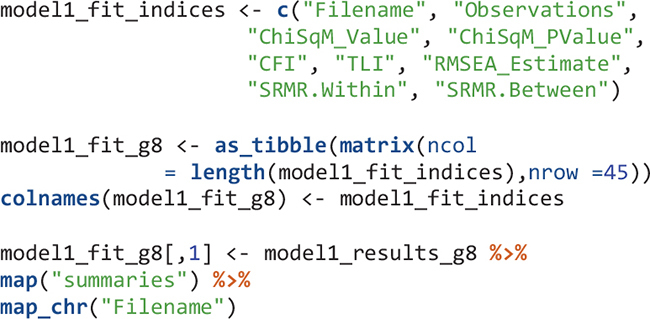



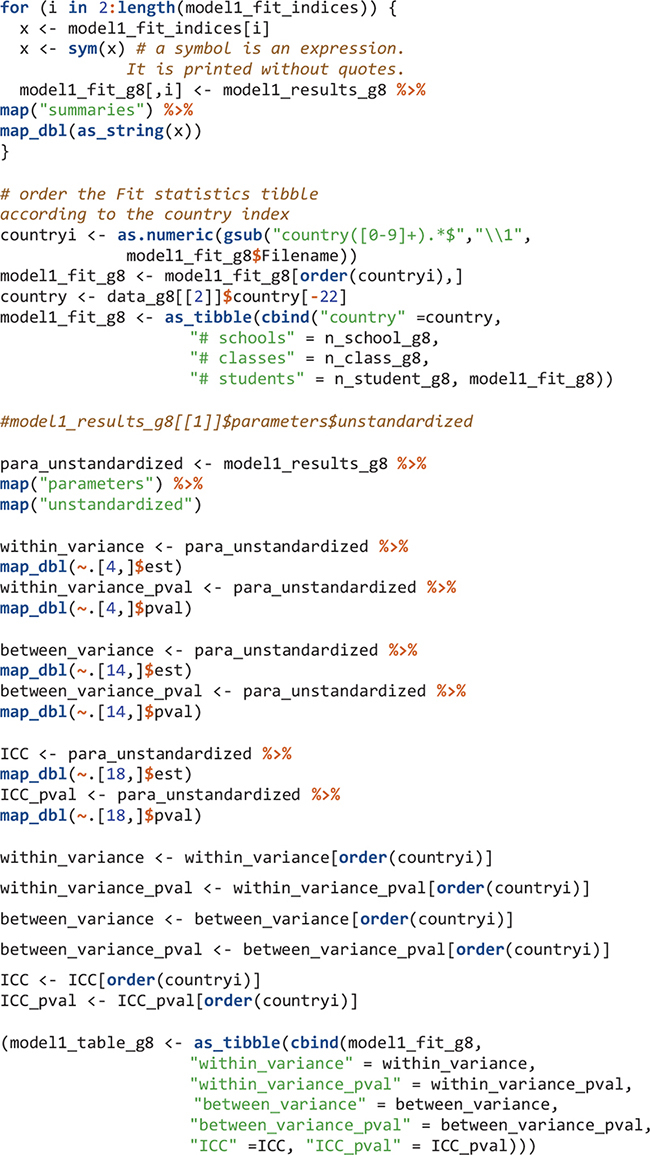


For eighth-grade Model 2, the flow is similar: create an object that contains Mplus input syntax sections, create Mplus input files, run the model in Mplus, output the data and Mplus output files, read the Mplus output files, and extract summary and parameter information from the Mplus output files.


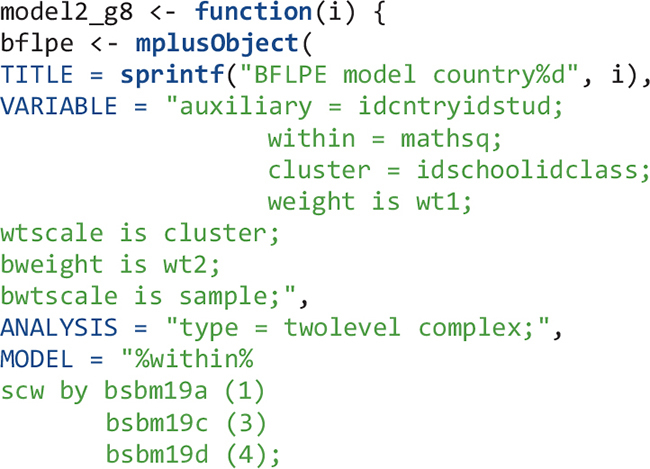



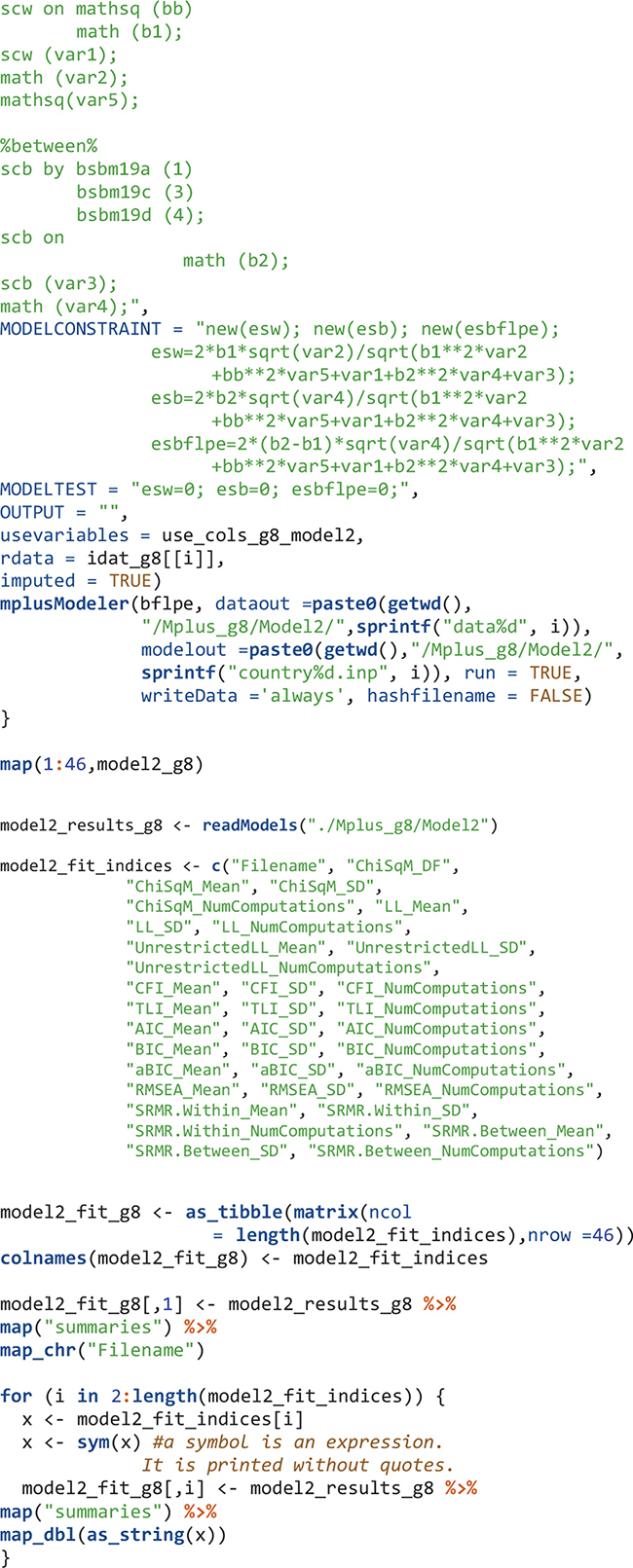



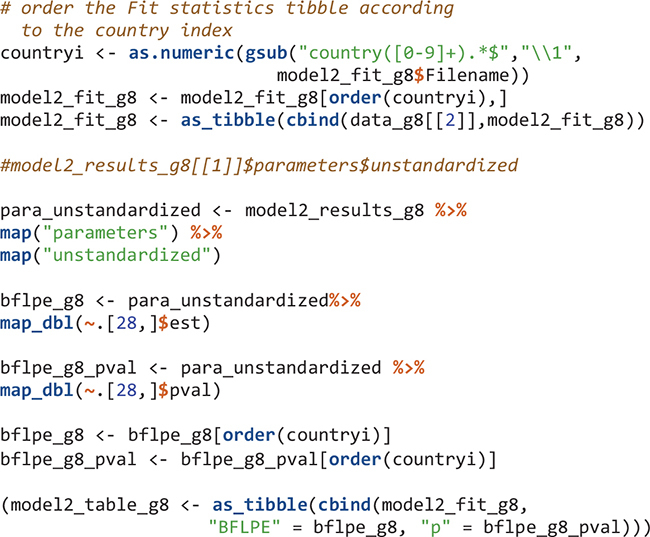


## Results

### Multilevel CFA Model (Model 1)

As explained in the Syntax section, Model 1 did not run successfully for Saudi Arabia (country47) in fourth grade and Jordan (country22) in eighth grade. In both cases, Mplus output messages that the Fisher information matrix is non-positive definite and this could be due to issues with starting values for the model parameters. Non-positive definite matrices cause problems in parameter estimation of latent variable modeling (i.e., Heywood cases; see Kolenikov and Bollen, 2012), indicate lack of model fit, and could be the result of model misspecification, empirical under-identification, sampling fluctuations, or even outliers (Bollen, 1987). For parameter estimation of multilevel CFA modeling with the maximum likelihood estimation with robust standard errors (MLR), by default, Mplus uses fixed starting values. These fixed starting values could lead to non-convergence of parameter estimation. For the two problematic cases (Saudi Arabia in fourth grade and Jordan in eighth grade) of Model 1, I manually modified the Mplus input files to use 10 random sets of starting values to address the issue of non-convergence of the fixed starting value run. After the modifications, the model estimation terminated normally, although there was a warning messaging of a non-positive definite covariance matrix for the latent variables.

In addition, after examining the summary results, I decided that the model for Saudi Arabia in eighth grade needed further attention because the estimates for the within-level variance and the between-level variance were both zero. This could suggest a problem with parameter estimation and changing starting values *may* solve the problem. I manually modified the Mplus input file to use 10 random sets of starting values. After the modification, the results were more trustworthy (see Table 1, row “Saudi Arabia*”).

Model 1 has two degrees of freedom. The model fit indices are in Table 1 for fourth grade and in Table 2 for eighth grade. Based on the regular model fit cutoffs (root mean square error of approximation, or RMSEA < 0.08; comparative fit index, or CFI > 0.95, Tucker–Lewis index, or TLI > 0.95) (Browne and Cudeck, 1993; Hu and Bentler, 1999; Kline, 2016), the model did not fit data from four (Czechia, France, Italy, and Saudi Arabia) of the 56 countries in fourth grade. However, only Italy had relatively poor model fit (CFI = 0.93, TLI = 0.79, RMSEA = 0.108). Czechia, France, and Saudi Arabia had relatively low TLI (0.93, 0.91, and 0.90, respectively) but their CFI values are greater than 0.95 and RMSEA values less than 0.08. In eighth grade, two (England and Saudi Arabia) out of the 46 countries did not have good model fit. Nevertheless, although both countries had relatively low TLI values (0.93 and 0.94, respectively), their CFI (0.98 for both countries) and RMSEA values (0.075 and 0.037, respectively) indicated adequate model fit.

Tables 1, 2 also include the within-level variance, the between-level variance, and the intraclass correlation coefficient (ICC) of mathematics self-concept for each country. For the three datasets (fourth grade Saudi Arabia, eighth grade Saudi Arabia, and eighth grade Jordan) that needed random sets of starting values, the estimates of the within-level variance and the between-level variance were negative. For the other datasets, in fourth grade, the within-level variance was statistically significant at the 0.05 level for all countries except Jordan (*p* = 0.326) and Oman (*p* = 0.777); the between-level variance was statistically significant at the 0.05 level for all countries except Australia (*p* = 0.071), Czechia (*p* = 0.090), Ireland (*p* = 0.088), and Netherlands (*p* = 0.430), as well as for Jordan (*p* = 0.379), and Oman (*p* = 0.781); the ICC ranged from 0.4% (Netherlands) to 27.7% (Indonesia). In eighth grade, the within-level variance was statistically significant at the 0.05 level for all countries except Bahrain (*p* = 0.166), Egypt (*p* = 0.279), and Morocco (*p* = 0.472); the same three countries had statistically non-significant between-level variance (*p*-Values were 0.194, 0.330, and 0.479 for Bahrain, Egypt, and Morocco, respectively); the ICC ranged from 1.8% (Japan) to 23.9% (England). A small ICC means that there is little between class variation compared to within class. However, a small ICC of mathematics self-concept can also be viewed as resulting from social comparison largely within the class.

### Model 2 (The BFLPE Model)

Model 2 has nine degrees of freedom. Tables 3, 4 show modeling results for fourth grade and eighth grade, respectively. For each model fit index, there is a mean and a standard deviation. This is because the model for each country in each grade was actually run five times using the five plausible values of mathematics achievement in the TIMSS 2015 database and therefore there were five values for each model fit index. The mean and standard deviation of the five values were reported in the Mplus output. For example, the mean of CFI values for Abu Dhabi, United Arab Emirates in eighth grade was 0.93 with a standard deviation of 0.005. Using regular model fit cutoffs of CFI > 0.95 and TLI > 0.95 for the mean, most countries did not have adequate model fit. Using RMSEA < 0.08 for the mean, 52 out of 56 countries had good model fit in fourth grade and 43 out of 46 countries had good model fit in eighth grade.

**TABLE 3:** Results of Model 2 in Fourth Grade.

	χ^2^		CFI		TLI		RMSEA		BFLPE	
					
Country	Mean	*SD*	Mean	*SD*	Mean	*SD*	Mean	*SD*	Est.	*p*
Abu Dhabi, United Arab Emirates	121.49	12.74	0.77	0.012	0.59	0.022	0.050	0.003	–0.570	<0.001
Buenos Aires, Argentina	115.45	6.88	0.90	0.004	0.81	0.008	0.043	0.001	–0.439	<0.001
Dubai, United Arab Emirates	52.26	2.79	0.94	0.003	0.90	0.005	0.025	0.001	–0.462	<0.001
United Arab Emirates	218.97	6.37	0.86	0.003	0.75	0.005	0.033	0.000	–0.620	<0.001
Armenia	100.91	9.00	0.91	0.007	0.85	0.013	0.043	0.002	–0.558	<0.001
Australia	33.75	6.02	0.98	0.003	0.97	0.006	0.021	0.002	–0.527	<0.001
Belgium (Flemish)	37.60	8.84	0.99	0.003	0.98	0.005	0.024	0.004	–0.618	<0.001
Bulgaria	104.37	5.98	0.91	0.004	0.85	0.007	0.050	0.002	–0.474	<0.001
Bahrain	157.59	10.69	0.74	0.010	0.53	0.019	0.044	0.002	–0.157	0.037
Canada	76.26	6.60	0.98	0.002	0.96	0.003	0.025	0.001	–0.398	<0.001
Chile	187.95	28.67	0.88	0.013	0.79	0.024	0.064	0.005	–0.523	<0.001
ON, Canada	71.35	6.99	0.97	0.003	0.94	0.004	0.039	0.002	–0.343	<0.001
QC, Canada	9.67	2.97	1.00	0.002	1.00	0.006	0.006	0.006	–0.324	<0.001
Cyprus	174.09	15.59	0.90	0.007	0.82	0.012	0.067	0.003	–0.264	<0.001
Czechia	140.08	15.09	0.95	0.005	0.90	0.010	0.053	0.003	–0.492	<0.001
Germany	61.01	5.34	0.98	0.002	0.96	0.004	0.038	0.002	–0.447	<0.001
Denmark	62.62	9.34	0.98	0.004	0.96	0.008	0.040	0.004	–0.291	<0.001
England	49.02	4.60	0.97	0.003	0.95	0.006	0.033	0.002	–0.423	<0.001
Spain	288.50	35.02	0.84	0.014	0.72	0.025	0.063	0.004	–0.482	<0.001
Finland	116.52	11.10	0.96	0.003	0.93	0.006	0.049	0.002	–0.315	<0.001
France	39.01	1.89	0.98	0.001	0.97	0.002	0.026	0.001	–0.505	<0.001
Georgia	209.74	22.49	0.70	0.019	0.46	0.034	0.075	0.004	–0.253	0.022
Hong Kong SAR	39.42	4.93	0.97	0.004	0.95	0.007	0.031	0.003	–0.602	<0.001
Croatia	118.68	5.83	0.94	0.003	0.90	0.005	0.055	0.001	–0.402	<0.001
Hungary	349.04	23.86	0.88	0.006	0.79	0.010	0.087	0.003	–0.717	<0.001
Indonesia	250.71	23.81	0.71	0.016	0.48	0.028	0.057	0.003	–0.136	0.193
Ireland	113.65	18.64	0.94	0.009	0.90	0.016	0.052	0.005	–0.340	<0.001
Iran, Islamic Rep. of	551.97	45.96	0.54	0.021	0.18	0.037	0.087	0.004	–0.635	<0.001
Italy	66.41	10.25	0.96	0.007	0.92	0.012	0.038	0.004	–0.508	<0.001
Jordan	98.30	10.00	0.76	0.021	0.57	0.037	0.035	0.002	0.064	0.631
Japan	47.61	5.33	0.99	0.002	0.97	0.004	0.031	0.002	–0.266	<0.001
Kazakhstan	45.19	5.16	0.95	0.007	0.91	0.013	0.029	0.002	–0.615	<0.001
Korea, Rep. of	149.84	15.52	0.95	0.004	0.92	0.008	0.058	0.003	–0.150	0.014
Kuwait	55.70	1.73	0.81	0.008	0.67	0.015	0.027	0.000	–0.130	0.213
Lithuania	79.85	13.86	0.95	0.009	0.91	0.017	0.042	0.004	–0.645	<0.001
Morocco	90.19	7.67	0.82	0.012	0.67	0.021	0.029	0.001	–0.483	<0.001
Northern Ireland	119.45	24.41	0.93	0.011	0.88	0.020	0.062	0.007	–0.334	<0.001
Netherlands	150.36	10.73	0.94	0.003	0.89	0.006	0.059	0.002	–0.444	<0.001
Norway (4th grade)	85.32	15.96	0.94	0.011	0.90	0.019	0.045	0.005	–0.124	0.049
Norway	72.50	12.62	0.96	0.006	0.93	0.011	0.040	0.004	–0.314	<0.001
New Zealand	224.77	15.70	0.89	0.005	0.81	0.008	0.062	0.002	–0.619	<0.001
Oman	109.48	7.07	0.68	0.019	0.43	0.034	0.035	0.001	–0.068	0.388
Poland	347.90	18.55	0.88	0.007	0.79	0.012	0.089	0.002	–0.340	<0.001
Portugal	108.94	13.21	0.95	0.005	0.91	0.009	0.049	0.003	–0.390	<0.001
Qatar	166.81	9.98	0.75	0.011	0.55	0.019	0.058	0.002	–0.596	<0.001
Russian Federation	69.99	9.92	0.97	0.005	0.94	0.008	0.037	0.003	–0.699	<0.001
Saudi Arabia	81.79	1.99	0.70	0.012	0.47	0.021	0.043	0.001	–0.213	0.176
Singapore	201.17	21.47	0.92	0.007	0.86	0.013	0.057	0.003	–0.288	<0.001
Serbia	135.18	19.78	0.88	0.014	0.78	0.025	0.059	0.004	–0.491	<0.001
Slovak Republic	156.41	14.81	0.91	0.004	0.84	0.008	0.053	0.003	–0.760	<0.001
Slovenia	138.55	13.50	0.93	0.006	0.88	0.011	0.057	0.003	–0.372	<0.001
Sweden	118.68	20.53	0.93	0.010	0.87	0.018	0.054	0.005	–0.333	<0.001
Turkey	162.33	16.64	0.88	0.007	0.78	0.013	0.051	0.003	–0.667	<0.001
Chinese Taipei	261.43	35.20	0.89	0.007	0.81	0.012	0.081	0.006	–0.275	<0.001
United States	151.87	15.46	0.95	0.004	0.92	0.007	0.040	0.002	–0.436	<0.001
South Africa	347.18	145.98	0.49	0.219	0.09	0.390	0.058	0.011	–1.167	<0.001

*CFI, comparative fit index; TLI, Tucker–Lewis index; RMSEA, root mean square error of approximation; BFLPE, big-fish-little-pond effect.*

**TABLE 4:** Results of Model 2 in Eighth Grade.

	χ^2^		CFI		TLI		RMSEA		BFLPE	
					
Country	Mean	*SD*	Mean	*SD*	Mean	*SD*	Mean	*SD*	Est.	*p*
Abu Dhabi, United Arab Emirates	67.40	4.80	0.93	0.005	0.88	0.008	0.037	0.002	–0.852	<0.001
Buenos Aires, Argentina	89.03	11.90	0.93	0.008	0.87	0.015	0.052	0.004	–0.586	<0.001
Dubai, United Arab Emirates	35.98	1.69	0.98	0.001	0.96	0.002	0.022	0.001	–0.696	<0.001
United Arab Emirates	107.50	2.64	0.96	0.001	0.93	0.002	0.025	0.000	–0.871	<0.001
Armenia	96.43	6.22	0.95	0.004	0.90	0.007	0.044	0.002	–0.332	<0.001
Australia	117.31	9.11	0.97	0.002	0.95	0.003	0.034	0.001	–0.569	<0.001
Bahrain	97.39	7.86	0.88	0.006	0.79	0.011	0.045	0.002	–0.410	<0.001
Botswana	460.69	39.79	0.69	0.018	0.45	0.033	0.092	0.004	–0.400	<0.001
Canada	371.04	40.56	0.94	0.005	0.90	0.008	0.068	0.004	–0.511	<0.001
Chile	199.85	18.59	0.92	0.005	0.86	0.009	0.066	0.003	–0.752	<0.001
ON, Canada	117.48	22.45	0.97	0.006	0.95	0.010	0.051	0.005	–0.376	<0.001
QC, Canada	50.93	6.64	0.98	0.002	0.97	0.003	0.034	0.003	–0.420	<0.001
Egypt	165.21	11.65	0.73	0.007	0.52	0.013	0.047	0.002	–0.161	0.044
England	45.45	3.95	0.97	0.002	0.95	0.004	0.029	0.002	–0.907	<0.001
Georgia	165.56	24.32	0.87	0.015	0.77	0.026	0.065	0.005	–0.427	<0.001
Hong Kong SAR	86.79	13.45	0.96	0.006	0.93	0.011	0.045	0.004	–0.876	<0.001
Hungary	159.88	11.51	0.96	0.002	0.92	0.004	0.058	0.002	–0.790	<0.001
Ireland	129.76	14.83	0.96	0.003	0.93	0.006	0.053	0.003	–0.491	<0.001
Iran, Islamic Rep. of	186.54	14.18	0.91	0.006	0.84	0.011	0.057	0.002	–0.582	<0.001
Israel	128.14	11.52	0.94	0.005	0.89	0.010	0.049	0.002	–0.602	<0.001
Italy	72.52	10.77	0.98	0.002	0.97	0.004	0.040	0.003	–0.552	<0.001
Jordan	201.46	8.40	0.76	0.006	0.57	0.010	0.052	0.001	–0.346	<0.001
Japan	104.93	6.71	0.97	0.001	0.95	0.003	0.047	0.002	–0.554	<0.001
Kazakhstan	83.12	8.78	0.95	0.005	0.91	0.009	0.041	0.003	–0.491	<0.001
Korea, Rep. of	159.41	22.59	0.97	0.003	0.94	0.005	0.056	0.004	–0.197	<0.001
Kuwait	77.87	8.01	0.88	0.009	0.79	0.017	0.041	0.002	–0.540	<0.001
Lebanon	66.35	7.66	0.90	0.012	0.82	0.021	0.040	0.003	–0.381	<0.001
Lithuania	50.63	3.43	0.98	0.001	0.97	0.002	0.033	0.001	–0.477	<0.001
Morocco	297.04	14.03	0.80	0.006	0.65	0.010	0.050	0.001	–0.288	<0.001
Malta	21.56	1.25	0.99	0.001	0.98	0.002	0.019	0.001	–0.756	<0.001
Malaysia	584.05	58.27	0.68	0.018	0.43	0.032	0.081	0.004	–0.756	<0.001
Norway (8th grade)	134.86	20.14	0.97	0.005	0.94	0.009	0.054	0.004	–0.319	<0.001
Norway	46.93	7.15	0.99	0.002	0.98	0.003	0.030	0.003	–0.265	<0.001
New Zealand	111.00	7.18	0.96	0.002	0.93	0.004	0.037	0.001	–0.871	<0.001
Oman	142.96	5.95	0.86	0.004	0.75	0.008	0.041	0.001	–0.122	0.056
Qatar	87.93	1.93	0.90	0.004	0.83	0.006	0.040	0.000	–0.663	<0.001
Russian Federation	53.84	3.52	0.98	0.001	0.97	0.002	0.032	0.001	–0.682	<0.001
Saudi Arabia	114.39	7.42	0.83	0.010	0.69	0.017	0.056	0.002	–0.166	0.064
Singapore	34.34	4.32	0.99	0.001	0.99	0.002	0.021	0.002	–1.317	<0.001
Slovenia	30.38	5.69	0.99	0.002	0.99	0.003	0.023	0.003	–0.395	<0.001
Sweden	112.72	14.63	0.97	0.004	0.94	0.007	0.053	0.004	–0.482	<0.001
Thailand	158.42	7.17	0.87	0.005	0.77	0.008	0.051	0.001	–0.717	<0.001
Turkey	122.98	2.32	0.96	0.001	0.93	0.002	0.046	0.000	–0.670	<0.001
Chinese Taipei	585.72	21.32	0.92	0.002	0.86	0.003	0.106	0.002	–0.404	<0.001
United States	98.12	7.25	0.98	0.001	0.97	0.002	0.031	0.001	–0.568	<0.001
South Africa	70.93	3.56	0.95	0.002	0.91	0.003	0.023	0.001	–1.046	<0.001

*CFI, comparative fit index; TLI, Tucker–Lewis index; RMSEA, root mean square error of approximation; BFLPE, big-fish-little-pond effect.*

In fourth grade, the BFLPE was negative and statistically significant at the 0.05 level in all but five countries (Indonesia, Jordan, Kuwait, Oman, and Saudi Arabia), ranging from −0.124 (Norway) to −1.167 (South Africa) with a mean of −0.461 and a median of −0.447 measured as the Cohen’s *d*. In eighth grade, the BFLPE was negative and statistically significant at the 0.05 level in all but two countries (Oman and Saudi Arabia), ranging from −0.161 (Egypt) to −1.317 (Singapore) with a mean of −0.576 and a median of −0.553. The model fit indices in general were not as good as those for Model 1. It is interesting to see that the countries where the BFLPE did not manifest tended to have some of the worst model fit.

## Discussion

Large-scale assessments such as those available from NCES are rich data sources for researchers to study substantive research questions. One particular challenge for using such data is due to their sizes. The researcher needs to navigate various documents and datasets to identify variables and information that are useful and has to be good at data wrangling. When the analysis has to be scaled up for many groups (e.g., states, countries, regions), manually running analysis for individual groups is tedious and should be avoided. Data science tools can be particularly useful because they can automate repeated actions.

In this study, I showed how to use three R packages, EdSurvey, MplusAutomation, and tidyverse to conduct a large-scale analysis of the BFLPE across countries. Mainly, the EdSurvey was used to obtain data, MplusAutomation was used to run complex multilevel latent variable models and to extract results from Mplus outputs, and tidyverse was used for data management. Although each of the three packages is quite useful in its own way, the combination of them is a powerful toolkit for applied quantitative researchers interested in using NCES data. With these few packages learned, a researcher can do most data wrangling and analysis of LSAs.

Other R packages have been developed that may also be useful for researchers interested in analysis of LSAs. The lavaan.survey package (Oberski, 2014) combines special features of the lavaan and survey packages to allow for SEM analysis of complex survey data. However, it also has some of the same limitations as lavaan and survey. For example, missing data cannot be handled with full information maximum likelihood together with survey weights. The MplusAutomation package, because it calls and therefore has the same capacity of modeling as Mplus, can apply more advanced methods to deal with missing data, complex survey designs, and other analysis issues. It is possible to only use existing R packages without having to rely on the external Mplus software to address the missing data and other issues. For example, the semTools package (Jorgensen et al., 2020) has the runMI function that can fit a lavaan model to multiply imputed datasets or fit the lavaan model while imputing the missing values using the Amelia (Honaker et al., 2011) or the mice (van Buuren and Groothuis-Oudshoorn, 2011) package. For more experienced R users, exploring various packages for specific data and analysis issues may be a joyful learning journey. However, for less experienced users who are interested in applying latent variable modeling to large-scale educational assessment data, I recommend spending time to get familiar with the three packages discussed in this study: EdSurvey, MplusAutomation, and tidyverse.

The BFLPE was found in 51 of the 56 countries in fourth grade and 44 of the 46 countries in eighth grade for the subject of mathematics, suggesting generalizability of the effect. Earlier work using TIMSS (Wang, 2015; Wang and Bergin, 2017) and PISA (Marsh and Hau, 2003; Seaton et al., 2009) also showed the existence of the BFLPE in many countries. While the theoretical explanation of the BFLPE is social comparison, it is not clear how students compare. Huguet et al. (2009) argued that forced upward social comparison with the entire class underlies the BFLPE and found that controlling for perceived relative standing would eliminate the BFLPE; however, Wang (2015); Wang and Bergin (2017) found that students’ perceived relative standing in the class did not eliminate but instead may decrease the BFLPE. All of these studies used cross-sectional data. In fact, the majority of existing BFLPE research has used cross-sectional, self-reported data. There is a need for future research based on alternative data types and formats of data collection.

Interestingly, Oman and Saudi Arabia did not have statistically significant BFLPE in both grades. Four (Cyprus, Algeria, Morocco, and Slovenia) of the 49 countries in Wang (2015) and one (Syrian Arab Republic) of the 59 countries in Wang and Bergin (2017) did not show statistically significant BFLPE for eighth-grade mathematics. It is not clear why these countries differed from other countries. It could be due to their education or social systems but a closer look at these countries may shed light on BFLPE research.

While the research on model fit of SEM is still quite active, there is little research on how these fit indices behave in large-scale analysis of complex survey data. In this project, I used traditional cutoffs for model fit indices that were developed based on single-level analysis and with the maximum likelihood estimator. The multilevel CFA model seemed to fit the data well in most of the countries, but the BFLPE model fit rather poorly in the majority of the countries. For the BFLPE model, the measurement model at both levels is saturated and constrained to have cross-level measurement invariance as in the multilevel CFA model. If the model fit indices are to be trusted, the poor fitting of the BFLPE model could be due to: (a) unmodeled relationships between the residuals of the self-concept indicator items and mathematics achievement, (b) the orthogonality assumption across levels, or (c) both (a) and (b). In SEM, it is typically not advised to include covariances between a predictor and the residuals of indicators of an outcome variable. The orthogonality assumption across levels is not a testable assumption for latent variables (mathematics self-concept in this project). Another possibility is that the relationship between mathematics achievement and mathematics self-concept could be reciprocal. While the BFLPE research uses achievement as the predictor and self-concept as the outcome, one’s self-concept could likely affect achievement.

Despite the large sample sizes, the structure of data used in this project is “simple” and data collection was through surveys only. The data are well organized and the unit of analysis is students. Data management is necessary for statistical modeling but could be done using techniques that are designed for traditional data analysis. A related concept is “big data,” which is a broader concept and the massive amount of data may be unstructured and in different formats such as texts, speeches, and photographs. From the “big data” standpoint, the data used for this study are “small data” – data that can be represented in spreadsheets on a single computer (Chen and Wojcik, 2016). In this study, I used many “small data” files, therefore, the term “large-scale.”

The use of technology allows the collection of behavior data that were not possible before. For example, the 2017 NAEP was administered for the first time as a digitally based assessment. Response process data were collected that could provide insights into students’ test-taking behaviors, how such behaviors relate to achievement, and even diagnostics of learning strategies. Other types of data such as videos, texts, online social network data (e.g., Twitter and Facebook) are additional examples. Researchers in psychology and other social sciences can take advantage of these more “novel” data types with the use of data science and big data tools (Chen and Wojcik, 2016).

This study shows that the analysis of many similarly structured datasets can be automated using data science tools. However, the researcher still needs to scrutinize modeling results to identify possible problems. Any result that looks suspicious should be examined more closely. For this project, I found that the initial results for Saudi Arabia in eighth grade could be problematic due to the estimates of variances of latent variables. Because the model did run and fit information could be extracted, I might have trusted the initial results. However, a closer look rendered that the initial model had a problem with starting values. The convenience of data science tools should not be substituted for content expertise.

This study has a didactic nature and focuses on analysis of LSAs. The field of data science and big data has begun to attract more researchers in social sciences (Gilmore, 2016); there is a high demand of tutorials showing “how to” use various data tools. Some tools are more general for writing purposes. For example, R Markdown is a powerful tool to create fully reproducible documents, combining code, results, explanatory texts, tables, references, etc. Other tools, such as those used in this study, are for more specific purposes. Teaching researchers how to use these tools can be a particularly useful area in its own right. We need “twofers” who can help bridge data engineering and domain knowledge to move both worlds forward.

## Data Availability Statement

Publicly available datasets were analyzed in this study. This data can be found here: https://timssandpirls.bc.edu/timss2015/international-database.

## Ethics Statement

Ethical review and approval was not required for the study on human participants in accordance with the local legislation and institutional requirements. Written informed consent from the participants’ legal guardian/next of kin was not required to participate in this study in accordance with the national legislation and the institutional requirements.

## Author Contributions

ZW conceived the study, performed the statistical analysis, and wrote the manuscript.

## Conflict of Interest

The author declares that the research was conducted in the absence of any commercial or financial relationships that could be construed as a potential conflict of interest.
